# Rapid Detection of ESBL-Producing *Enterobacteriaceae* in Blood Cultures

**DOI:** 10.3201/eid2103.141277

**Published:** 2015-03

**Authors:** Laurent Dortet, Laurent Poirel, Patrice Nordmann

**Affiliations:** Institut National de la Santé et de la Recherche Médicale, Le Kremlin–Bicêtre, France (L. Dortet, L. Poirel, P. Nordmann);; University of Fribourg, Fribourg, Switzerland, Department of Medicine (L. Poirel, P. Nordmann);; Hôpital Fribourgeois-hôpital Cantonal, Fribourg (P. Nordmann)

**Keywords:** Resistance, bacteria, antibiotic, antibacterial, gram-negative, β-lactam, cephalosporins, cephamycins, carbapenems, ESBL, Nordmann/Dortet/Poirel test, Enterobacteriaceae, MALDI-TOF, mass spectrometry, rapid diagnostic test

## Abstract

We rapidly identified extended-spectrum β-lactamase (ESBL) producers prospectively among 245 gram-negative bacilli–positive cultured blood specimens using the Rapid ESBL Nordmann/Dortet/Poirel test and direct bacterial identification using matrix-assisted laser desorption/ionization time-of-flight mass spectrometry. This combination identified ESBL-producing *Enterobacteriaceae* within 30 min and had high predictive values.

An essential parameter for improving the outcome of sepsis is early implementation of appropriate antibiotic therapy (*1*–*5*). Recently, using matrix-assisted laser desorption ionization time-of-flight (MALDI-TOF) mass spectrometry (MS) technology directly with blood cultures was found to help guide clinical management of bacteremia caused by gram-negative bacteria (GNB) (*6*). Resistance to broad-spectrum cephalosporins is spreading rapidly among *Enterobacteriaceae*, mostly related to acquisition of extended-spectrum β-lactamases (ESBLs) (*7*). ESBL-producing *Enterobacteriaceae* (ESBL-E) are usually resistant to most β-lactams except cephamycins and carbapenems.

Using PCR-based molecular techniques on positive blood cultures has been proposed for rapid identification of ESBLs (*8*); however, trained personnel and expensive material are required for their use. In addition, for the TEM- and SHV-type enzymes, detailed gene sequence analysis is required for differentiating narrow-spectrum β-lactamases from ESBLs.

Rapid identification of ESBL producers is possible by using the ESBL Nordmann/Dortet/Poirel (NDP) test (*9*), which is based on the biochemical detection of the hydrolysis of the β-lactam ring of cefotaxime (a broad-spectrum cephalosporin). Presence of these bacteria has previously been evaluated with cultured bacteria and with spiked blood cultures (*9*).

In this study, we evaluated the ESBL NDP test prospectively in clinical settings directly from blood cultures. Identification of the bacterial species was done concomitantly from blood cultures by using enhanced MALDI-TOF procedures.

## The Study

During November 2012–May 2013, we studied a single blood culture positive for GNB from each of 245 patients hospitalized at the Bicêtre hospital, a 950-bed hospital located in a suburb of Paris. Positivity of blood cultures was detected by using the BacT/Alert system (bioMérieux, La Balme-les-Grottes, France). After obtaining Gram stain results, we tested the blood cultures positive for GNB directly for 1) ESBL-E by using the ESBL NDP test, and 2) species identification by using the MALDI-TOF MS technique.

We adapted the protocol of the ESBL NDP test for detection of the ESBL-E from blood cultures (*9*) (Technical Appendix). The detailed MALDI-TOF MS protocol using the VITEK MS system (bioMérieux) is described in Detailed Methods in the Technical Appendix.

We performed antibiotic susceptibility testing (AST) by the disk diffusion method using bacterial colonies grown from blood cultures according to the Clinical Laboratory Standards Institute (CLSI) recommendations (*10*). The same MALDI-TOF technology and the API Gram negative Identification product (bioMérieux) were used for confirmatory identification of bacteria. AST results, obtained 48 h after blood cultures were identified as positive, were interpreted according to the CLSI breakpoints, as updated in 2014 (*10*). MIC of cefotaxime, ceftazidime, and cefepime were determined on Muller-Hinton (MH) agar and MH agar supplemented with 4 μg/mL of tazobactam (final concentration).

We used the double-disk synergy test (DDST) for the phenotypic detection of ESBL producers (*11*), according to the CLSI recommendations. For each sample, 1 disk contained cefotaxime, ceftazidime, or cefepime, and a second disk contained ticarcillin and clavulanate (*10*). The DDST was also performed on MH agar plates (bioMérieux) containing cloxacillin (150 mg/L) to inhibit cephalosporinase activity of natural producers of those inducible cephalosporinases. Because the DDST was performed in parallel to the AST, results were obtained 48 h later. The DDST was considered to be the reference standard for the detection of ESBL-E.

We also used molecular biology techniques to identify the ESBL genes. We used PCR to amplify DNA, which we then sequenced using *bla*_TEM_, *bla*_SHV_, and *bla*_CTX-M_ primers (*9*).

During the study, 245 blood specimens that were collected from patients hospitalized in any unit of the hospital were cultured and grew GNB (Table 1). The 245 cases of bacteremia were attributed to *Enterobacteriaceae* (211, 86.1%), nonfermentative GNB (31, 12.7%), and anaerobic GNB (3, 1.2%) (Table 2). Three blood cultures (1.2%) were positive for 2 enterobacterial species (Table *2*). *Escherichia coli* was the predominant enterobacterial species (118/211, 55.9%); the next most prevalent were *Klebsiella pneumoniae* (37/211, 17.5%) and *Enterobacter cloacae* (20/211, 9.5%). *Pseudomonas aeruginosa* (24/31, 77.4%) was the predominant non-fermentative GNB (Table 2). Anaerobic GNB belonged to the *Bacteroides fragilis* group (*Bacillus fragilis* and *Bacillus vulgatus*).

**Table 1:** Origin of gram-negative bacilli identified from blood cultures using rapid detection methods in bacterial blood cultures*

Hospital department	No. gram-negative bacilli	No. (%) *Enterobacteriaceae*					No. (%) nonfermenting bacilli
		Total	*Escherichia coli*	Other	ESBL-negative	ESBL-positive	
Cardiology	5	4 (80)	2 (50)	2 (50)	4 (100)	0	1 (20)
Digestive surgery	9	8 (89)	6 (75)	2 (25)	6 (75)	2 (25)	1 (11)
Orthopedic surgery	3	3 (100)	2 (67)	1 (33)	3 (100)	0	0
Endocrinology	2	2 (100)	0	2 (100)	2 (100)	0	0
Gerontology	9	7 (78)	3 (43)	4 (57)	7 (100)	0	2 (22)
Gynecology-obstetric	3	2 (67)	0	2 (100)	2 (100)	0	1 (33)
Hepato-gastroenterology	20	18 (90)	8 (44)	10 (56)	15 (83)	3 (17)	2 (10)
Emergency	40	37 (93)	30 (81)	7 (19)	32 (86)	5 (14)	3 (8)
Infectious diseases	28	25 (89)	16 (64)	9 (36)	17 (68)	8 (32)	3 (11)
Nephrology	28	21 (75)	8 (38)	13 (62)	11 (52)	10 (48)	7 (25)
Neurology	8	8 (100)	5 (63)	3 (38)	8 (100)	0	0
Pediatric unit	2	2 (100)	2 (100)	0	2 (100)	0	0
Pneumology	4	3 (75)	2 (67)	1 (33)	2 (67)	1 (33)	1 (25)
Intensive care unit	67	57 (85)	24 (42)	33 (58)	42 (74)	15 (26)	10 (15)
Rhumatology	3	2 (67)	2 (100)	0	1 (50)	1 (50)	1 (33)
Urology	14	12 (86)	8 (67)	4 (33)	10 (83)	2 (17)	2 (14)
Total	245	211 (86)	118 (56)	93 (44)	164 (78)	47 (22)	34 (14)

**Table 2:** Results of MALDI-TOF and extended-spectrum β-lactamase NDP testing of 245 blood cultures positive for gram-negative bacilli and definitive identification and antibiotic resistance phenotype obtained with cultured bacteria*

30 min				24–48 h	
MALDI-TOF identification	No.	ESBL NDP test	Definitive identification	β-Lactam resistance phenotype a
*Enterobacteriaceae*					
* Escherichia coli*	38	–		*E. coli*	Wild-type
	47	–		*E. coli*	Penicillinase
	1	–		*E. coli*	IRT
	1	–		*E. coli*	Low-level cephalosporinase
	1	–		*E. coli*	Overexpressed cephalosporinase
	30	+		*E. coli*	ESBL
* Proteus mirabilis*	3	–		*P. mirabilis*	Penicillinase
* Salmonella* spp.	1	–		*Salmonella* Typhimurium	Wild-type
	1	–		*Salmonella* Enteritidis	Wild-type
	1	–		*Salmonella* Paratyphi A	Wild-type
	1	–		*Salmonella* Typhi	Wild-type
	1	–		*Salmonella* Kentucky	Penicillinase
* Klebsiella pneumoniae*	23	–		*K. pneumoniae*	Wild-type
	1	–		*K. pneumoniae*	Acquired cephalosporinase
	13	+		*K. pneumoniae*	ESBL
* K. oxytoca*	5	–		*K. oxytoca*	Wild-type
* Citrobacter koseri*	2	–		*C. koseri*	Wild-type
* Enterobacter cloacae*	12	–		*E. cloacae*	Wild-type
	5	–		*E. cloacae*	Overexpressed cephalosporinase
	3	+		*E. cloacae*	ESBL
* Enterobacter aerogenes*	5	–		*E. aerogenes*	Wild-type
* Citrobacter braaki*	1	–		*C. braaki*	Wild-type
* C. freundii*	1	+		*C. freundii*	ESBL
* Morganella morganii*	4	–		*M. morganii*	Wild-type
* Providencia rettgeri*	2	–		*P. rettgeri*	Wild-type
* Proteus vulgaris*	1	–		*P. vulgaris*	Wild-type
* Serratia marcescens*	4	–		*S. marcescens*	Wild-type
None	1	–		*E. coli* + *Proteus mirabilis*	Penicillinase + Penicillinase
	2	–		*K. pneumoniae* + *S. marcescens*	Wild-type + Penicillinase
Nonfermenting bacilli					
* Pseudomonas aeruginosa*	24	–		*Pseudomonas aeruginosa*	Ceftazidime S
* Acinetobacter baumannii*	2	–		*A. baumannii*	Ceftazidime S
* A. junii*	1	–		*A. junii*	Ceftazidime S
* Stenotrophomonas maltophilia*	2	–		*Stenotrophomonas maltophilia*	Ceftazidime S
* Roseomonas gilardii*	1	–		*Roseomonas gilardii*	Ceftazidime S
* Sphingobacterium multivorum*	1	–		*Sphingobacterium multivorum*	Ceftazidime S
Anaerobic gram-negative bacilli					
* Bacteroides fragilis*	1	–		*B. fragilis*	Wild-type
	1	–		*B. fragilis*	Cefotaxime R
* B. vulgatus*	1	–		*B. vulgatus*	Cefotaxime R
*MALDI-TOF, matrix-assisted laser desorption/ionization time-of-flight; NDP, Nordmann/Dortet/Poirel test; ESBL, extended spectrum β-lactamase; –, negative test result; IRT, inhibitor-resistant TEM β-lactamase; +, positive test result.					

We identified bacteria directly from blood culture using the MALDI-TOF technique for 237 (96.7%) isolates; results corresponded to bacterial identification after culture (Table 2). *Salmonella* spp. (n = 5) were correctly identified at the genus level (Table 1). For the 3 positive blood cultures that contained 2 enterobacterial species (Table 2), results were noninterpretable.

ESBL-E producers (n = 47) represented 22.3% of *Enterobacteriaceae*. Among the 47 ESBL-E, 30 *E. coli*, 13 *K. pneumoniae*, 3 *E. cloacae*, and 1 *Citrobacter freundii* were identified from patients who were infected in the community or the hospital (detailed data not shown) (Table 1; Table 3). Most of the ESBLs were of the CTX-M-type (49/51, 96.1%); CTX-M-15 was predominant (35/51, 68.6%). The proportion of ESBL producers were 35.1%, 25.4%, and 15% among *K. pneumoniae*, *E. coli*, and *E. cloacae*, respectively (Table 3). The ESBL NDP test perfectly identified the 47 ESBL-E pathogens (Tables 1, 3). Accordingly, a 100% correlation between intermediated susceptibility of resistance to cefotaxime and positivity of the ESBL NDP test was observed, whereas this correlation was of 76.6% and 74.4%, respectively, when ceftazidime and cefepime susceptibility results were used for this same comparison (Table 3). The ESBL NDP test gave negative results for 164 specimens that were negative for ESBL-E (Table 2). The ESBL NDP test revealed a cefotaxime-hydrolyzing enzyme that was not inhibited by tazobactam for 1 *K. pneumoniae* isolate that produced an acquired cephalosporinase, 3 of the 5 *E. cloacae* that overproduced chromosome-encoded AmpC, and 2 *Bacteroides* spp. strains (data not shown).

**Table 3:** MC values of several β-lactams and β-lactamase content for the extended-spectrum b-lactamase –producing *Enterobacteriaceae* in blood cultures*

Hospitalization unit ^a^	Species	MICs (μg/mL)†							β-Lactamase content ^c^		
		CAZ	CTX	FEP	CAZ/TZB	CTX/TZB	FEP/TZB		SHV	TEM	CTX-M
Emergency	*Escherichia coli*	**2†**	8	**2**	0.19	0.032	0.023		–	–	**CTX-M-1**
Rhumatology	*E. coli*	**1**	12	**2**	0.19	0.032	0.016		–	TEM-1	**CTX-M-1**
Emergency	*E. coli*	32	48	8	0.75	0.25	0.064		**SHV-12‡**	–	**CTX-M-3**
Emergency	*E. coli*	**1.5**	16	6	0.38	0.094	0.064		–	TEM-1	**CTX-M-14**
ICU	*E. coli*	**1**	16	3	0.19	0.016	0.023		–	TEM-1	**CTX-M-14**
Nephrology	*E. coli*	**0.5**	12	**1.5**	0.094	0.047	0.016		–	TEM-1	**CTX-M-14**
Nephrology	*E. coli*	**0.38**	8	**1.5**	0.125	0.032	0.023		–	TEM-1	**CTX-M-14**
Surgery	*E. coli*	**0.5**	12	**2**	0.19	0.032	0.032		–	TEM-1	**CTX-M-14**
ICU	*E. coli*	8	96	6	0.19	0.032	0.032		–	–	**CTX-M-15**
ICU	*E. coli*	**1**	16	**0.75**	0.094	0.032	0.047		–	–	**CTX-M-15**
Infectious diseases	*E. coli*	12	128	12	0.25	0.094	0.064		–	–	**CTX-M-15**
Infectious diseases	*E. coli*	16	128	12	0.19	0.094	0.094		–	–	**CTX-M-15**
Surgery	*E. coli*	12	128	12	0.38	0.5	2		–	–	**CTX-M-15**
Urology	*E. coli*	16	192	16	0.25	0.19	0.064		–	TEM-1	**CTX-M-15**
Urology	*E. coli*	12	192	8	0.25	0.125	0.064		–	TEM-1	**CTX-M-15**
Infectious diseases	*E. coli*	32	128	16	0.19	0.047	0.064		–	TEM-1	**CTX-M-15**
Nephrology	*E. coli*	48	6	**1**	0.38	0.19	0.094		**SHV-12**	TEM-1	–
Nephrology	*E. coli*	48	6	**1**	0.38	0.19	0.094		**SHV-12**	TEM-1	–
Infectious diseases	*E. coli*	12	128	8	0.19	0.047	0.023		–	–	**CTX-M-15**
Nephrology	*E. coli*	16	128	12	0.25	0.094	0.047		–	–	**CTX-M-15**
ICU	*E. coli*	256	256	192	32	24	2		–	–	**CTX-M-15**
ICU	*E. coli*	8	96	6	0.19	0.064	0.064		–	–	**CTX-M-15**
ICU	*E. coli*	12	128	6	0.125	0.064	0.125		–	–	**CTX-M-15**
Infectious diseases	*E. coli*	32	256	24	0.5	0.19	0.094		–	–	**CTX-M-15**
Infectious diseases	*E. coli*	32	256	24	0.5	0.19	0.094		–	–	**CTX-M-15**
Infectious diseases	*E. coli*	256	256	16	0.5	0.016	0.094		–	–	**CTX-M-15**
Pneumology	*E. coli*	32	256	16	0.5	0.094	0.032		–	TEM-1	**CTX-M-15**
Emergency	*E. coli*	256	12	**1**	0.094	0.032	0.016		–	–	**CTX-M-27**
ICU	*E. coli*	**1**	12	**1**	0.094	0.023	0.023		–	–	**CTX-M-27**
Nephrology	*E. coli*	**2**	2	**1.5**	0.094	0.016	0.016		–	TEM-1	**CTX-M-55**
ICU	*Klebsiella pneumoniae*	**3**	32	4	0.125	0.023	0.032		–	TEM-1	**CTX-M-15**
Infectious diseases	*K. pneumoniae*	32	256	8	0.38	0.125	0.094		–	TEM-1	**CTX-M-15**
ICU	*K. pneumoniae*	128	256	64	1	0.25	0.19		LEN-5	–	**CTX-M-15**
ICU	*K. pneumoniae*	256	256	32	2	0.5	2		LEN-5	TEM-1	**CTX-M-15**
Nephrology	*K. pneumoniae*	16	128	6	0.25	0.064	0.047		SHV-1	TEM-1	**CTX-M-15**
Hepatology	*K. pneumoniae*	6	96	6	0.19	0.047	0.19		SHV-1	TEM-1	**CTX-M-15**
Nephrology	*K. pneumoniae*	8	96	4	0.25	0.064	0.032		SHV-1	TEM-1	**CTX-M-15**
ICU	*K. pneumoniae*	48	256	24	0.5	0.064	0.064		SHV-1	TEM-1	**CTX-M-15**
Hepatology	*K. pneumoniae*	16	64	6	0.5	0.25	0.125		SHV-11	TEM-1	**CTX-M-15**
Hepatology	*K. pneumoniae*	12	48	6	0.5	0.19	0.094		SHV-11	TEM-1	**CTX-M-15**
ICU	*K. pneumoniae*	12	128	12	0.38	0.047	0.094		SHV-11	TEM-1	**CTX-M-15**
Nephrology	*K. pneumoniae*	48	256	16	0.5	0.19	0.5		SHV-11	TEM-1	**CTX-M-15**
ICU	*K. pneumoniae*	256	256	24	0.25	0.047	0.064		SHV-28	TEM-1	**CTX-M-15**
ICU	*Enterobacter cloacae*	96	256	32	1	1	0.25		–	–	**CTX-M-15**
Nephrology	*E. cloacae*	12	192	12	0.25	0.125	0.047		–	TEM-1	**CTX-M-15**
ICU	*E. cloacae*	12	128	6	0.25	0.047	0.047		SHV-1	TEM-1	**CTX-M-15**
Emergency	*Citrobacter freundii*	16	12	**2**	2	1	0.5		–	**TEM-3** +TEM-1	**CTX-M-55**

ICU, intensive care unit, CAZ, ceftazidime; CTX, cefotaxime; FEP, cefepime; TZB, tazobactam; –, negative PCR result. †Numbers in bold text indicate MIC values reported in the range of susceptibility according to Clinical Laboratory Standards Institute guidelines as updated in 2014 (susceptible means MIC ≤1 μg/mL, ≤4 μg/mL and ≤2 μg/mL for cefotaxime, ceftazidime and cefepime, respectively). ‡Bold, underlined text indicates ESBL names.

The ESBL NDP test used with blood cultures had a sensitivity of 100% (95% CI: 92.4%–100%), a specificity of 100% (95% CI: 97.7%–100%), a positive predictive value of 100% (95% CI: 99.2%–100%) and a negative predictive value of 100% (95% CI: 97.8%–100%) for the detection of ESBL-E.

## Conclusions

Detection of ESBLs that are the main source of cephalosporin resistance in *Enterobacteriaceae* still relies on antibiotic susceptibility testing, results of which usually take 24–48 h. We show that the ESBL NDP test directly performed on positive blood cultures is a reliable technique to identify ESBL-E within 30 min. Although these results are promising, they should be further confirmed in other countries where the prevalence and the epidemiology of ESBL-E might be different. A strong correlation between intermediate susceptibility or resistance to cefotaxime and positivity of the ESBL NDP test was observed (Table 3). Similar correlation between resistance to cefotaxime and ESBL production in *Enterobacteriaceae* was obtained in the United States (*12*). A concomitant use of the Carba NP test (*13*) directly from blood culture will also identify carbapenemase producers (such as *K. pneumoniae* carbapenemase producers) that also confer clavulanic-acid–inhibited resistance to cephalosporins.

This inexpensive ESBL NDP test might be implemented worldwide. It may optimize rapid choices of antibiotics for treating bloodstream infections. It may also contribute to avoidance of overuse of carbapenems. Finally, a rapid detection of ESBL-E coupled with bacterial species identification will enhance identification of ESBL in species likely to be the source of nosocomial outbreaks (*K. pneumoniae*, *Enterobacter* spp.) and facilitate implementation of a rapid strategy for containment (*14*).

Technical Appendix Overview and detailed description of methods used for rapid detection of ESBL-producing *Enterobacteriaceae* in blood cultures.
