# Supplementary material for: Rapid Detection of ESBL-Producing Enterobacteriaceae in Blood Cultures
Source: Emerg Infect Dis. 2015 Mar;21(3):504–7. doi: 10.3201/eid2103.141277 (PMC4344277; doi:10.3201/eid2103.141277)
Supplement: Technical Appendix — Overview and detailed description of methods used for rapid detection of ESBL-producing Enterobacteriaceae in blood cultures. [file 14-1277-Techapp-s1.pdf]

Article DOI: http://dx.doi.org/10.3201/eid2103.141277

Rapid Detection of ESBL-Producing *Enterobacteriaceae* in Blood Cultures

Methods Overview

MALDI-TOF MS bacterial identification directly on positive blood culture samples and definitive identification of bacterial species

One milliliter of blood culture was transferred into an 1.5-mL Eppendorf tube. Fifty microliters of Triton 10% was added in the tube that was then briefly vortexed. After a centrifugation step at 13,000 × *g* for 2 min, the supernatant was discarded and the bacterial pellet was resuspended in 500 µL of distilled water. After a second centrifugation step at 13,000 × *g* for 2 min, the supernatant was discarded and the bacterial pellet was resuspended into 50 µL of 100% ethanol (Thermo Scientific, Villebon-sur-Yvette, France). One drop of this suspension was spotted in duplicate on the disposable plate. The dried spots were overlaid with 1 µL of the matrix solution (VITEK-MS CHCA; bioMérieux, La Balme-les-Grottes, France) and air-dried for 1–2 min at room temperature, as recommended by the manufacturer. The loaded slide was then inserted into the VITEK MS system. Spectra were analyzed and identifications were calculated automatically by the advanced spectrum classifier algorithm provided by the manufacturer. Definitive identification retained as the gold standard was obtained with cultured bacteria using the MALDI-TOF MS bacterial identification (VITEK-MS; bioMérieux) and the Api20E biochemical gallery (bioMérieux).

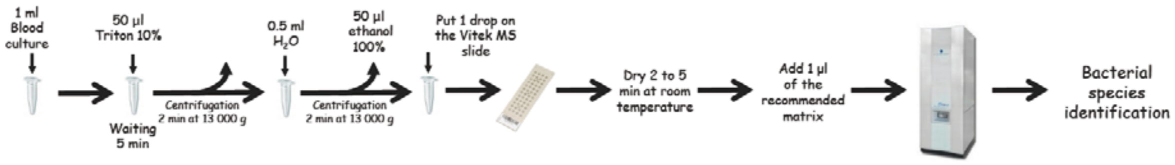

Detailed Methods

Rapid Detection of ESBL Activity in *Enterobacteriaceae*

ESBL NDP Test Directly from Blood Cultures

Protocol

1. Transfer 0.5 mL of *Enterobacteriaceae*–positive blood culture (using a syringe with needle) to each of 3 Eppendorf tubes, size 1.5 mL (tubes A, B, and C).
2. Add 50 µL of a solution of Triton 10% (vol/vol).
3. Vortex.
4. Incubate 5 min at room temperature.
5. Centrifuge at 13,000 × *g* for 2 min.
6. Discard the supernatant.
7. Resuspend the bacterial pellet in 500 µL of distilled water.
8. Check that bacterial colonies have been properly resuspended. If needed, mix up and down with a pipette.
9. Centrifuge at 13,000 × *g* for 2 min.
10. Discard the supernatant.
11. Resuspend the bacterial pellet in 100 µL of 20 mmol/L Tris-HCl lysis buffer (B-PERII, Bacterial Protein Extraction Reagent; Thermo Scientific, Pierce).
12. Check that bacterial colonies have been correctly resuspended. If necessary, mix up and down with a pipette.
13. Add 10 µL of a tazobactam concentrated solution (40 mg/mL) in the tube C.
14. Add (i) 100 µL of the revelation solution (Solution R) in the tube A and (ii) 100 µl Solution R + cefotaxime 6 mg/mL in the tubes B and C.
15. Incubate at 37°C for a maximum of 15 min.

16. Optical reading of the color of each tube.

Principle of the Test:

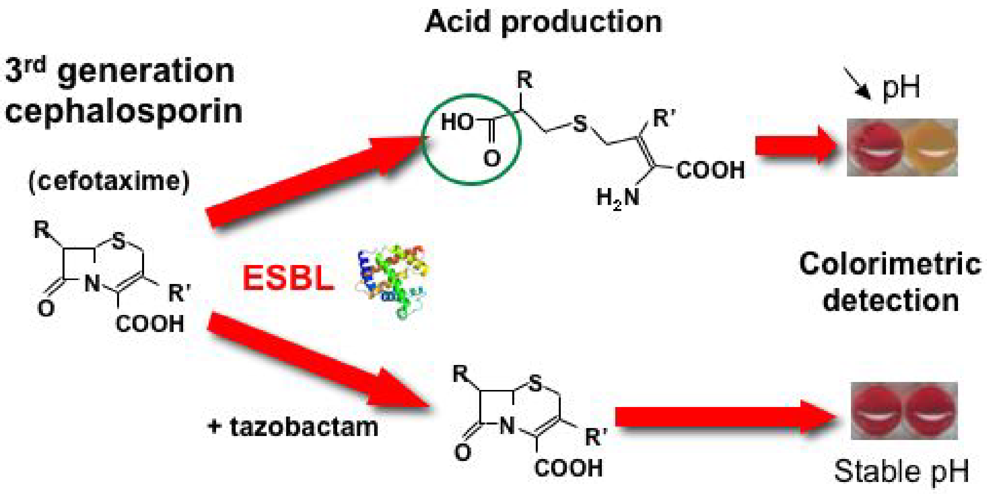

Interpretation:

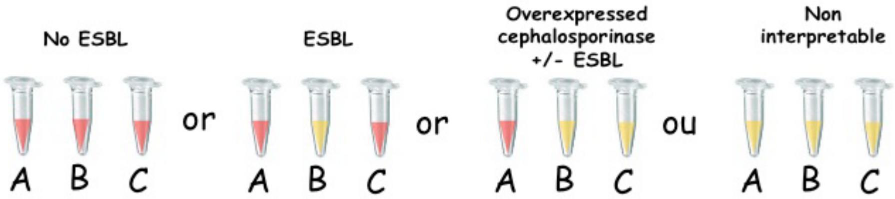

	No antibiotic (tube A)	Cefotaxime (tube B)	Cefotaxime + tazobactam (tube C)
No ESBL	Red	Red	Red
ESBL	Red	Orange/yellow	Red
Cephalosporinase or Cephalosporinase + ESBL	Red	Orange/yellow	Orange/yellow
Non interpretable	Yellow	Yellow	Yellow

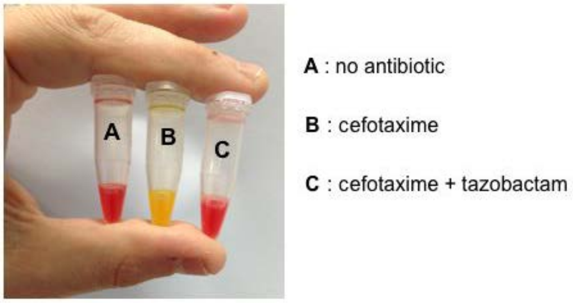

ESBL-producing *E. coli*

ESBL-producing *E. coli*. The *Enterobacteriaceae*–positive blood culture specimens contain A) no antibiotic; B) cefotaxime; C) cefotaxime and tazobactam.

Material

- 1.5-mL Eppendorf tubes
- Cefotaxime sodium salt (Sigma-Aldrich) or cefotaxime (drug used for patient treatment).
- Tazobactam sodium salt (Sigma-Aldrich, Cat: T-2820)
- B-PERII, Bacterial Protein Extraction Reagent (Thermo Scientific, Pierce), Cat: 78260.
- Concentrated solution of Triton X-100 (Sigma-Aldrich, Cat: T-8787)
- Negative (wild-type *E. coli*) and positive (*Klebsiella pneumoniae* CTX-M-15) controls.

Preparation and Storage of Solution R

1. Prepare a concentrated solution of red phenol 0.5% w/v
2. Mix 2 mL of the concentrated red phenol solution (strongly vortex before pipetting to resuspend the solution) in 16.6 mL of distilled water
3. Adjust the pH of the solution at a value 7.8 by adding drops of a NaOH solution (1 N)

Solution R is stable at room temperature for 1 week and may be kept at –20°C for several months.

Solution R + cefotaxime (6 mg/mL) has to be prepared extemporaneously.

However, batches of cefotaxime powders may be weighted in advance and kept at 4°C for 2 weeks if solution A is not added.

Preparation and Storage of Concentrated Tazobactam Solution (40 mg/mL)

1. Add 250 µL of distilled water in 10 mg of tazobactam sodium salt (Sigma-Aldrich, Cat: T-2820)

2. Prepare aliquots of 10 μL of this concentrated solution of tazobactam (40 mg/mL)

3. Those aliquots can be stored at −20°C for 1 month
